# Exploration of Binding Mechanism of a Potential *Streptococcus pneumoniae* Neuraminidase Inhibitor from Herbaceous Plants by Molecular Simulation

**DOI:** 10.3390/ijms21031003

**Published:** 2020-02-03

**Authors:** Shanshan Guan, Ketong Zhu, Yanjiao Dong, Hao Li, Shuang Yang, Song Wang, Yaming Shan

**Affiliations:** 1 College of Food Engineering, Jilin Engineering Normal University, Changchun 130052, Jilin, China; zhuketong@jlenu.edu.cn (K.Z.); dongyj1606@jlenu.edu.cn (Y.D.); lihao@jlenu.edu.cn (H.L.); yangshuang@jlenu.edu.cn (S.Y.); 2 Key Laboratory of Molecular Nutrition at Universities of Jilin Province, Changchun 130052, Jilin, China; 3 Laboratory of Theoretical and Computational Chemistry, Institute of Theoretical Chemistry, Jilin University, Changchun 130023, Jilin, China; ws@jlu.edu.cn; 4 National Engineering Laboratory for AIDS Vaccine, School of Life Sciences, Jilin University, Changchun 130012, Jilin, China

**Keywords:** *Streptococcus pneumoniae*, neuraminidase, chlorogenic acid, molecular docking, molecular dynamics simulation

## Abstract

*Streptococcus pneumoniae* can cause diseases such as pneumonia. Broad-spectrum antibiotic therapy for *Streptococcus pneumoniae* is increasingly limited due to the emergence of drug-resistant strains. The development of novel drugs is still currently of focus. Abundant polyphenols have been demonstrated to have antivirus and antibacterial ability. Chlorogenic acid is one of the representatives that has been proven to have the potential to inhibit both the influenza virus and *Streptococcus pneumoniae*. However, for such a potential neuraminidase inhibitor, the interaction mechanism studies between chlorogenic acid and *Streptococcus pneumoniae* neuraminidase are rare. In the current study, the binding mechanism of chlorogenic acid and *Streptococcus pneumoniae* neuraminidase were investigated by molecular simulation. The results indicated that chlorogenic acid might establish the interaction with *Streptococcus pneumoniae* neuraminidase via hydrogen bonds, salt bridge, and cation-π. The vital residues involved Arg347, Ile348, Lys440, Asp372, Asp417, and Glu768. The side chain of Arg347 might form a cap-like structure to lock the chlorogenic acid to the active site. The results from binding energy calculation indicated that chlorogenic acid had strong binding potential with neuraminidase. The results predicted a detailed binding mechanism of a potential *Streptococcus pneumoniae* neuraminidase inhibitor, which will be provide a theoretical basis for the mechanism of new inhibitors.

## 1. Introduction

*Streptococcus pneumoniae* (*S. pneumoniae*) is a kind of pathogen that can cause diseases such as pneumonia, otitis media, meningitis, and septicemia. This pathogen mainly infects children and the elderly people and has a very high fatality rate [1,2]. Broad-spectrum antibiotic therapy for *S. pneumoniae* is increasingly limited due to the emergence of drug-resistant strains. Therefore, the development of novel drugs is still currently of focus [3,4]. 

Neuraminidase are a key virulence factor, as they can remove sialic acid from host cell-surface glycans, probably unmasking certain receptors to facilitate bacterial adherence and colonization [5,6]. The neuraminidase of *S. pneumoniae* includes type A, B, and C, among which type A (NanA) has the strongest activity and best preservation [7]. NanA has a wide substrate specificity and cleaves α2,3-, α2,6-, and α2,8-linked sialic acids, whereas NanB and NanC show only considerable activity toward α2,3-linked substrates [8]. Due to NanA’s vital role in *S. pneumoniae* life cycle, it has emerged as an attractive target for the development of novel drugs [9].

Many pathogens possess neuraminidase, among which the influenza virus is the most representative one, with the exception of *S. pneumoniae*. Influenza virus neuraminidase (NA) is also a major surface glycoprotein of the virus. NA could cleave the terminal linkage of the sialic acid receptor, which results in the release of the progeny virions from the host cells [10]. It is reported that secondary infections with *S. pneumoniae* cause severe pneumonia and enhance lethality during influenza epidemics and pandemics, and *S. pneumoniae* NanA has been reported to contribute to this synergism by supporting viral release when added upon infection [11]. Influenza virus NA inhibitors have been widely developed and applied; however, by contrast, the NanA inhibitors of *S. pneumoniae* are not well studied. NA (from influenza virus) and NanA (from *S. pneumoniae*) have structural and functional similarities, which provide an opportunity for dual inhibitor design. The so-called dual inhibitors are those that inhibit both NA and NanA [9].

Recently, molecular docking and molecular dynamics simulation provided great assistance for modern drug development [12,13]. Software programs such as AutoDock Vina and Gromacs were widely used to search potential inhibitor for protein targets [14,15]. Previously, abundant herbaceous plants have been demonstrated to have antivirus and antibacterial ability according to the clinical data, and a number of polyphenols were regarded as active molecules. Chlorogenic acid is one of the main polyphenols, which can be found in herbs such as burdock, eucommia, honeysuckle, and wormwood [9,16]. 

Chlorogenic acid has been proven to have the potential to inhibit both influenza viruses and *S. pneumoniae* [16,17]. As a potential influenza virus NA inhibitor, the inhibition modes of chlorogenic acid have been generally studied [17,18]. However, the studies on the mechanism of interaction between chlorogenic acid and NanA are rare. In order to explore molecular inhibition mechanism of the potential NanA inhibitor, chlorogenic acid, molecular docking, molecular dynamics simulation and free energy calculation approach were applied in this study. The findings of this study might be useful for future exploration of efficient drug targets and provide theoretical insight into a new mechanism of *S. pneumoniae* inhibitors.

## 2. Results

This study explored via a series of computational methods. Three computational performances (molecular docking, molecular dynamics simulation, and free energy calculation) were undertaken. Firstly, molecular docking was applied to obtain the NanA–chlorogenic acid complex. Subsequently, the molecular dynamics simulation was performed to investigate the binding mode of chlorogenic acid and the dynamic behavior of the complex. After obtaining the stable simulated trajectory, the binding free energy was calculated to assess the binding potential of chlorogenic acid. The detailed research procedures is shown in the Materials and Methods section.

### 2.1. Analysis of Reliability of the Investigated Complex System

**Figure 1:**
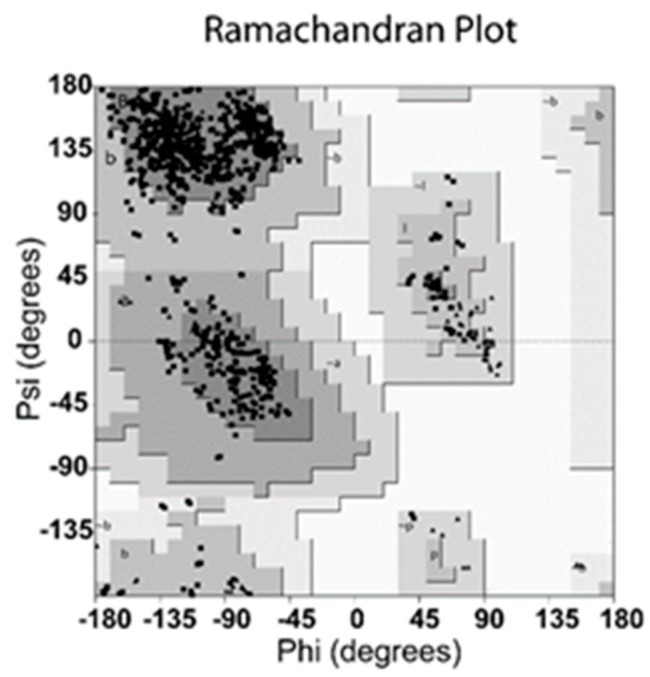
Ramachandran plot of optimized *Streptococcus pneumoniae* neuraminidase type A (NanA) protein system.

The validation was carried out using Ramachandran plot calculations computed with the Procheck program by examining the detailed residue-by-residue stereochemical quality of NanA structure before docking, and the result is shown in Figure 1. Altogether, 100% of the investigated residues were located in allowed regions, which validated the availability of the optimized NanA protein system [10,19].

**Figure 2:**
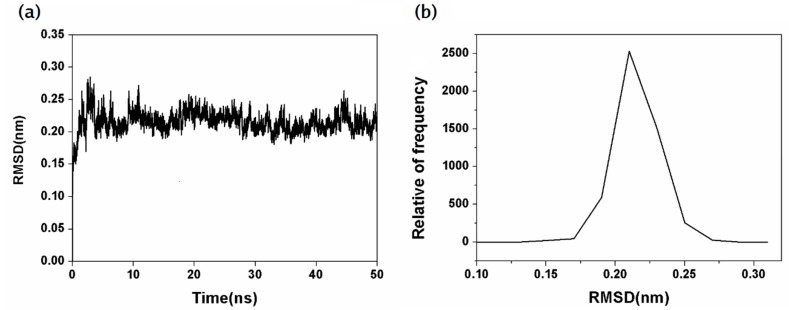
(**a**) Root-mean-square deviation (RMSD) plot of the NanA–chlorogenic acid complex during molecular dynamics simulation. (**b**) Average RMSD values for the system during the 50 ns molecular dynamics simulation.

After 50 ns simulation, the root-mean-square deviations (RMSD) of the backbone Cα atoms of the NanA was first investigated to evaluate if the complex system could reach equilibrium during the simulation [20]. As shown in Figure 2a,b, the RMSD curves of the NanA could be stabilized around 0.22 nm during in 50 ns, suggesting that the structure of the equilibrium stage could be applied to analyze the optimal binding mode between NanA and chlorogenic acid.

### 2.2. Detail Binding Mode of the NanA–Chlorogenic Acid Complex

**Figure 3:**
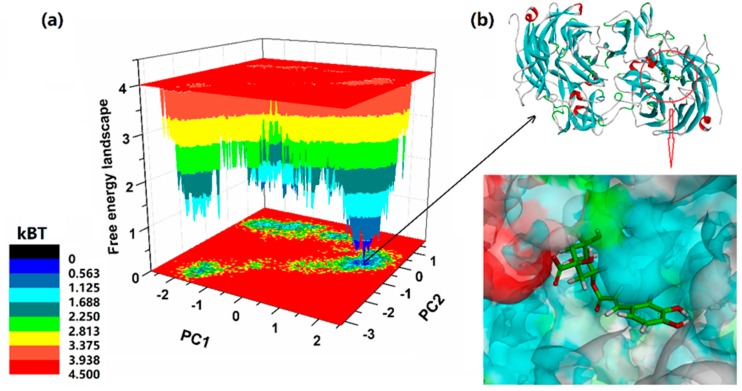
(**a**) Relative free energy surfaces along the first two principle components (PC1, PC2) of the NanA–chlorogenic acid complex. (**b**) Predicted integral binding poses of NanA–chlorogenic acid.

In order to obtain the most stable complex structure, cluster analyses of the NanA–chlorogenic acid complex were investigated to determine the optimal binding modes [20]. In the cluster analysis plot, the conformations found in the blue area indicated more stable and lower energy states than those found in the red area. In addition, these lower energy conformations extracted from blue areas generally could be chosen as the best analysis subjects for the binding modes [10]. The integral binding poses of NanA–chlorogenic acid based on the above analysis is shown in Figure 3. The results revealed that chlorogenic acid could be bound in the pocket located on the catalytic active center of NanA (this center was located in the pocket surrounded by Arg347, Asp364, Asp372, Asp417, Arg663, Arg721, and Tyr752) [21].

**Figure 4:**
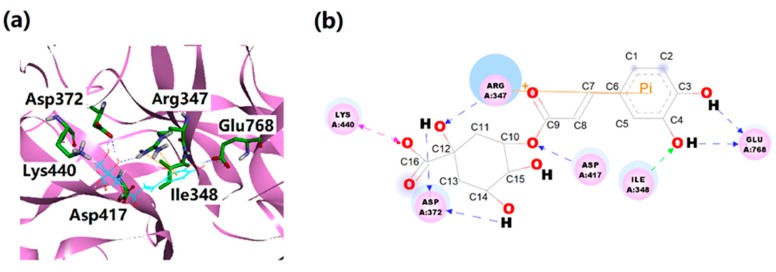
(**a**) Predicted detail binding modes of chlorogenic acid in the NanA–chlorogenic acid complex. (**b**) Two-dimensional interactions between NanA and chlorogenic acid.

The detailed binding mode of the complex based on the above analysis is shown in Figure 4. In Figure 4, the binding mode of the NanA–chlorogenic acid complex revealed that there are seven possible hydrogen bonds between NanA and chlorogenic acid. Specifically, the side chain of Arg347 established a hydrogen bond with the oxygen of C12 (chlorogenic acid). The main chain of Ile348 established a hydrogen bond with the oxygen of C4 (chlorogenic acid). The carboxyl group of the Asp372 established two hydrogen bonds with the oxygen of C12 and C14 (chlorogenic acid). The Asp417 and Glu768 were stabilized by their carboxyl groups with chlorogenic acid by one and two hydrogen bonds, respectively.

**Figure 5:**
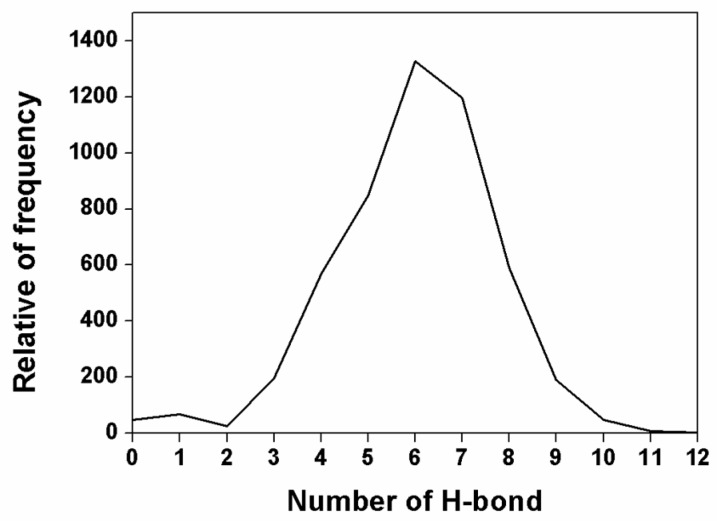
Distribution of hydrogen bond numbers between NanA and chlorogenic acid.

The predicted binding pose observed above was determined by evaluating the number of hydrogen bonds between NanA and chlorogenic acid. The probability of occurrence of hydrogen bonds was calculated by using the Gromacs program “gmx bond” [22]. As shown in Table 1, probability of occurrence of the mentioned hydrogen bonds was reasonably high, except that of Arg347, which suggests that these hydrogen bonds exist stably. The presence (%) of hydrogen bonds between chlorogenic acid and active site residues Arg347, Ile348, Asp372, Asp417, and Glu768 was 19.6%, 58.4%, 78.4%, 67.3%, and 99.9%, respectively. In addition, the distribution of hydrogen bond numbers between NanA and chlorogenic acid was calculated at the same time. As shown in Figure 5, the hydrogen bond numbers remained at 6–7 during the whole simulation, which was consistent with the above-mentioned hydrogen bond modes as well.

**Figure 6:**
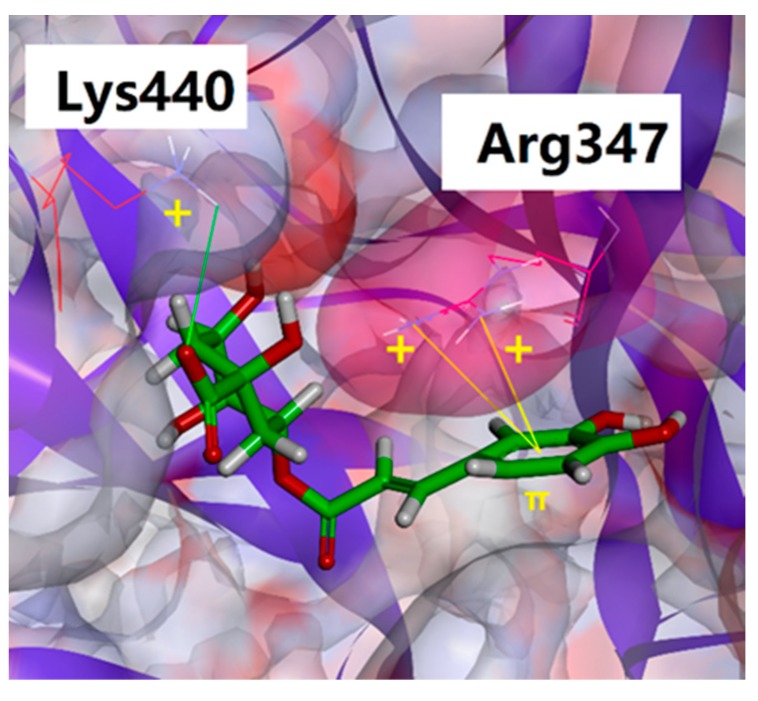
The detail interaction between chlorogenic acid and Arg347/Lys440.

Besides hydrogen bonds, salt bridge and cation-π interaction might also play vital roles in the complex. As shown in Figure 4 and Figure 6, the salt bridge was mainly formed between Lys440 and the carboxyl group of chlorogenic acid, and cation-π interaction involved Arg347 and benzene ring of chlorogenic acid. It is noteworthy that the side chain of Arg347 could form a cap-like structure to lock the benzene ring of chlorogenic acid into the active site of NanA.

**Figure 7:**
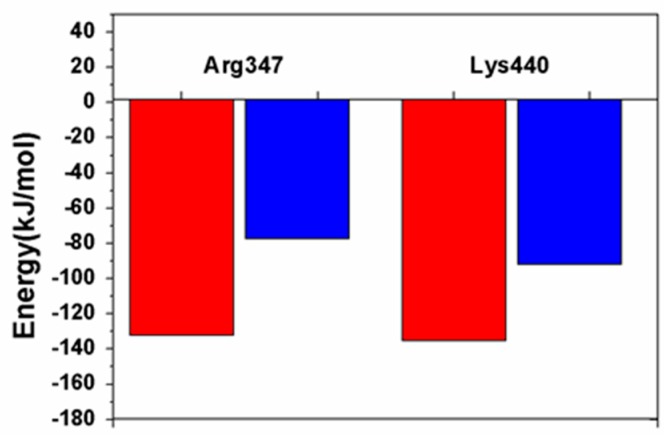
Binding energy contributions of Arg347 and Lys440.

The contributions of the two positive amino acids mentioned above that interacted with chlorogenic acid by salt bridge and cation-π were determined via energy calculation. As shown in Figure 7, the contribution of electrostatic interactions and van der Waals interactions (E_ele_ + E_vdw_, red columns) of Arg347 and Lys440 were obvious in the complex by lower values of −132.39 ± 2.37 kJ/mol and −135.15 ± 7.43 kJ/mol. Moreover, the value of total binding energy (blue columns) of Arg347 and Lys440 was −77.17 ± 0.82 kJ/mol and −91.87 ± 2.65 kJ/mol. These results indicate that Arg347 and Lys440 contribute to very high binding.

**Figure 8:**
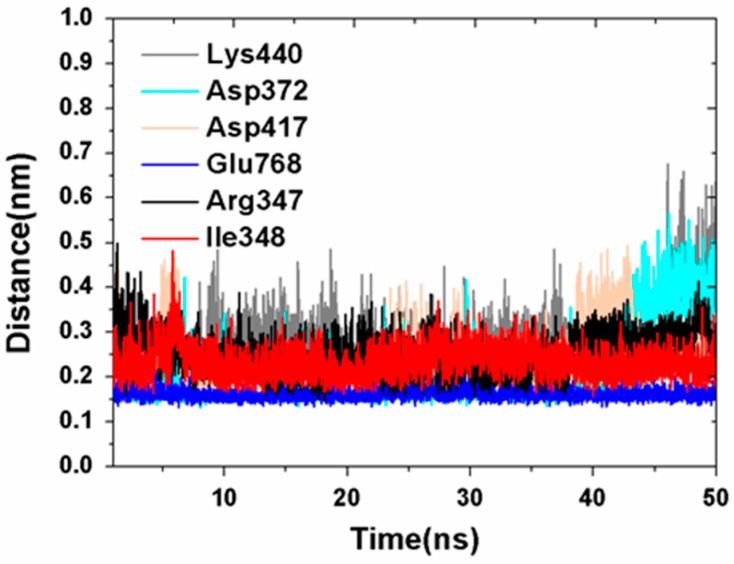
The distances between predicted residues of NanA and chlorogenic acid during 50 ns simulation.

In addition, the predicted interactions above were further determined by calculating the distance between NanA and chlorogenic acid. The distances between the predicted residues of NanA and chlorogenic acid were calculated by using the Gromacs program “gmx distance” [20]. The corresponding results are shown in Figure 8. As shown in Figure 8, all of the distances monitored were around 0.3–0.4 nm during the 50 ns simulation, which indicated the accuracy of the predicted binding pose.

### 2.3. Total Binding Energy of the NanA–Chlorogenic Acid Complex

The total binding energy of the complex system, an important standard measure of binding affinity between NanA and chlorogenic acid, was also calculated by “gmx mmpbsa”. As shown in Table 2, the value of total binding energy of the NanA–chlorogenic acid complex was −829.44 ± 19.31 kJ/mol, indicating that chlorogenic acid had strong binding interactions with NanA. In addition, the value of electrostatic energy was calculated at −953.77 ± 32.38 kJ/mol, indicating that electrostatic interactions were dominant in the total energy contribution. These results consist with the predicted binding modes in Section 2.2, in which the NanA established interaction with chlorogenic acid mainly via hydrogen bonds, salt bridge, and cation-π interaction. 

### 2.4. Comparing the Binding Modes of NanA Between Chlorogenic Acid and Zanamivir 

In this section, the interaction modes of NanA between chlorogenic acid and zanamivir, an effective influenza virus neuraminidase inhibitor [13], were compared. At the same time, the flexibility changes of the NanA induced by chlorogenic acid are explored.

It is reported that in the NanA–zanamivir complex, upon zanamivir binding, the cationic guanidinium group tightly interacts via a cluster of salt bridges formed with Asp372, Asp364, and Asp417, and the NanA loop in which Asp372 is located is pushed upwards/outwards with a maximal shift, whereas the side chain of Arg366 rotates away from the active site [5]. Nevertheless, in the NanA–chlorogenic acid complex of this study, due to the lack of positive charge groups, chlorogenic acid tended to form hydrogen bonds rather than salt bridges with Asp372 and Asp417 (as described in Section 2.2). The detail binding difference between negative groups of the NanA active site and zanamivir/chlorogenic acid can be found in Figure S1. 

**Figure 9:**
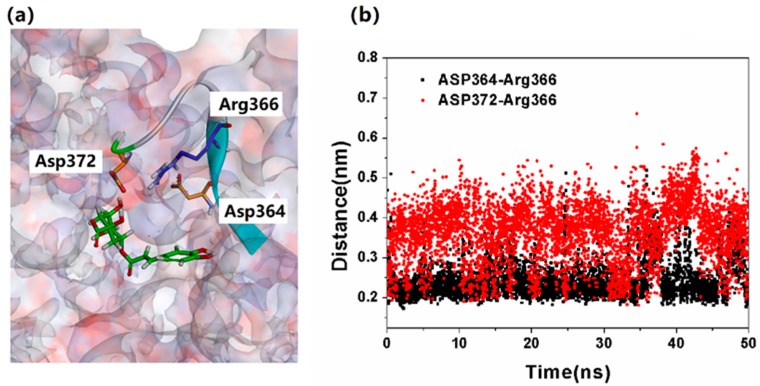
(**a**) The salt bridge between Arg366 and Asp372/Asp364. (**b**) The monitored distance between Arg366 and Asp372/Asp364.

In a previous study, a severe clash between the guanidinium groups of zanamivir and Arg366 was detected, which were attributed to the weak inhibitory potency of zanamivir, and the clash was able to break the salt bridge between Arg366 and Asp372/Asp364 [5]. However, In the NanA–chlorogenic acid complex, as shown in Figure 9a, the location of chlorogenic acid did not conflict with Arg366. The results can be proven by the stable salt bonds (between Arg366 and Asp372/Asp364), as shown in Figure 9b as well.

**Figure 10:**
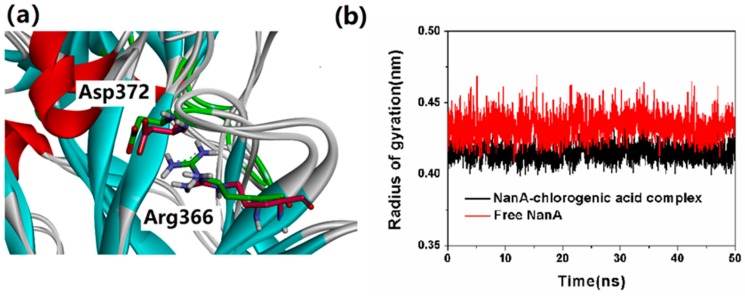
(**a**) The side chain direction of Asp372 and Arg366 in complex-NanA and free-NanA; (**b**) the radius of gyration of the loop in which Asp372 and Arg366 were located in complex-NanA and free-NanA.

In addition, the flexibility change of the loop in which Asp372 and Arg366 were located was investigated as well. As shown in Figure 10a, the binding of chlorogenic acid did not significantly change the side chain direction of Asp372 and Arg366. In order to further monitor the flexibility change of the loop in which Asp372 and Arg366 were located, the radius of gyration of the loop in complex-NanA and free-NanA were calculated. As shown in Figure 10b, the radius of gyration in complex-NanA did not change significantly during the whole simulation; moreover, the value of radius of gyration in complex-NanA was even lower than that in free-NanA. The results show that the combination of chlorogenic acid reduced the flexibility of the loop, indicating that chlorogenic acid had an effect on the loop, which might have been related to the hydrogen bonds detected in the binding mode.

**Figure 11:**
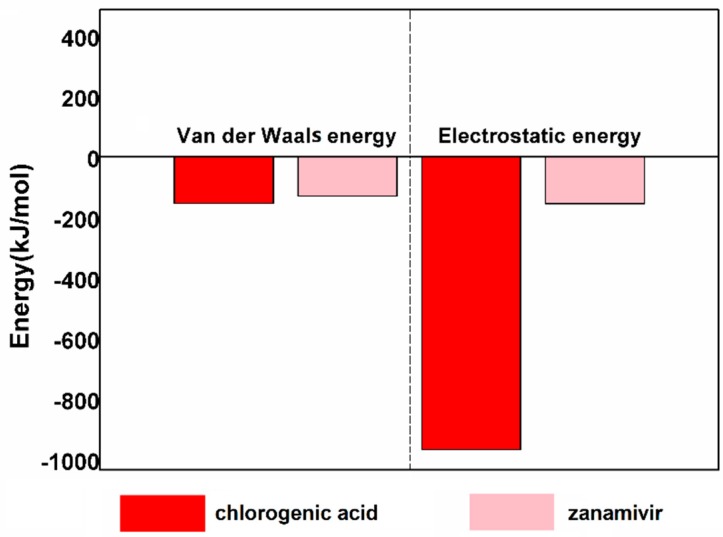
The difference of binding energy contribution from van der Waals and electrostatic energy in the NanA–chlorogenic acid and NanA–zanamivir complexes.

In terms of the binding free energy, the difference between the NanA–chlorogenic acid complex and the NanA–zanamivir complex were compared as well. As shown in Figure 11, the value of van der Waals energy between NanA with chlorogenic acid and zanamivir was −140.06 ± 15.42 kJ/mol and −115.91 ± 8.92 kJ/mol. In addition, the electrostatic energy contribution of chlorogenic acid and zanamivir was −953.77 ± 32.38 kJ/mol and −140.82 ± 68.28 kJ/mol. The results indicate that occupation of chlorogenic acid at the NanA active site was more favorable.

## 3. Discussion

The design of new *S. pneumoniae* drugs has always been a hot topic. NanA, as a new potential drug target with unique functions, is attracting the interest of scientists. Chlorogenic acid is one of the main polyphenols extracted from herbs, which have been demonstrated to have the ability to inhibit the function of the influenza virus and *S. pneumoniae*. Although many studies have focused on the influenza virus NA and chlorogenic acid, the interaction mechanism between *S. pneumoniae* and chlorogenic acid are still waiting to be clarified further theoretically. In this study, molecular simulation methods were applied to investigate the binding mechanism between *S. pneumoniae* NanA and chlorogenic acid. The results of this study indicated that chlorogenic acid was able to bond effectively with NanA through hydrogen bonding, salt bridge, and cation-π interaction, and the value of electrostatic energy was calculated at −953.77 ± 32.38 kJ/mol, indicating that electrostatic interactions were dominant in the total energy contribution. The results provide theoretical clues for chlorogenic acid inhibitory ability. 

It was found in the above analysis of the binding pattern that in the NanA–chlorogenic acid complex, the kinds of residues interacting with chlorogenic acid were partly similar with those interacting with zanamivir in the NanA–zanamivir complex [5]. However, the binding directions between both inhibitors are distinctly different. Zanamivir is an effective influenza virus neuraminidase inhibitor, and due to the structural and functional similarities between influenza virus NA and *S. pneumoniae* NanA, the NanA-zanamivir complex was crystallized, although zanamivir has been proven to own weak inhibitory potency to *S. pneumoniae* NanA [5]. In the reported study, a severe clash between the guanidinium groups of zanamivir and Arg366 (NanA) was detected; however, in the NanA–chlorogenic acid complex, the location of chlorogenic acid did not conflict with Arg366. In the recorded experiments, it seemed that a slight reduction in NanA activity, leading to reduced release of the biofilm and colonization, can have a dramatic effect on *S. pneumoniae* colonization. The results of this study indicated that the value of total binding energies of the NanA–chlorogenic acid complex was −829.44 ± 19.31 kJ/mol, indicating that chlorogenic acid has favorable binding interactions with NanA. The structural stability of NanA active site may be favorable for chlorogenic acid binding. Exploring the action mode of potential inhibitors is of benefit for the development of new antimicrobial drugs. 

## 4. Materials and Methods 

### 4.1. Preparation of Initial Complex

NanA from *S. pneumoniae* D39 was chosen for the preferred model to investigate the chlorogenic acid binding pose. The sequence of NanA is shown in Figure S2. The three-dimensional structure of NanA was obtained from the Research Collaboratory for Structural Bioinformatics Protein Data Bank (RCSB PDB ID: 3H72) [21]. All the structural members from crystal NanA were retained and the unstructured atoms, molecules, and crystal water were removed from the simulation system. The structure of chlorogenic acid was obtained from ZINC Data Bank (ZINC ID: ZINC2138728) [23], and the structure of chlorogenic acid is shown in Figure S3. Energy minimization was performed on NanA with Gromacs 5.1.4 using the steepest descent techniques before docking calculations [24,25]. The structural rationality of NanA was checked by carrying out Ramachandran plot calculations via the Procheck program [19,26].

### 4.2. Molecular Docking

Autodock Vina, a fast and accurate procedure to dock small compounds into fixed protein binding sites, was used for automatic placement of chlorogenic acid in the binding pockets of NanA to obtain the initial structure of the complex [24,27]. In order to facilitate the comparison, for the investigated complex in this study, only one monomer of the NanA was bound with chlorogenic acid and the other monomer was free. For AutoDock Vina, a grid of 26 × 26 × 26 points in the *x-*, *y-*, and *z*-axis directions was built with a grid spacing of 1Å, the center of which was treated as the active center of one monomer of NanA, and the exhaustiveness was set to 20 [10]. The grid detail used in the docking simulation is shown in Figure S4.

### 4.3. Molecular Dynamics Simulation

The complex system was subjected to molecular dynamics simulation in a periodic boundary condition using the Gromacs 5.1.4 software package with SPC (simple point charge) water model [25,28]. The Gromos 54 A7 force field was applied to describe both the NanA and chlorogenic acid [29]. The parameterization of the chlorogenic acid was produced by Automated Topology Builder (ATB) server [30]. Protonation states for titratable residues were determined at pH 6.5 by using PROPKA [31]. The results are shown in Table S1.

To keep the system at an electrically neutral state, 12 chloridions were added to randomly replace the water molecules. First, energies of the complex system were relaxed with steepest-descent energy minimization to eliminate steric clashes or incorrect geometry. Thereafter, 500ps NVT (constant number of particles, volume, and temperature) and NPT (constant number of particles, pressure, and temperature) were alternately operated with position restraints on NanA and chlorogenic acid to generate the relaxation of the solvent molecules in two phases. The solvent molecules were equilibrated with fixed protein at 310K, and the initial velocities were chosen from a Maxwellian distribution. Subsequently, the proteins and inhibitor were relaxed in a step-by-step manner and heated up to 310K [24]. The long-range electrostatics were described with the particle mesh Ewald algorithm with an interpolation order of 4, a grid spacing of 0.16 nm and the Coulomb cutoff distance of 1.0 nm [32]. The LINCS (linear constraint solver) algorithm was used to constrain all bonds [24]. Temperature and pressure coupling types were set with V-rescale and Parrinello–Rahman, respectively [33]. In the NVT ensemble, the temperature of the systems reached a plateau at the desired value (reference temperature = 310 K; time constant = 0.1 ps). In addition, the equilibration of pressure (reference pressure = 1.0 bar; time constant = 2.0 ps) was performed under the NPT ensemble. Finally, 50 ns molecular dynamics simulations for collecting data with a time step of 2 fs and coordinates saved every 2 ps were initiated [10,34]. A replicate of 50 ns MD of the complex was ran at the same time.

### 4.4. Binding Energy Calculation

Molecular mechanics Poisson–Boltzmann surface area (MM-PBSA) was applied as a scoring function in computational drug design to estimate the interaction-free energies in biomolecular interactions [35,36]. Using the “gmx mmpbsa”, the binding free energy of complex was calculated from 100 snapshots extracted from the equilibrium stage MD trajectory [37]. Furthermore, the binding energy was decomposed on a per residue basis to analyze the individual energy contributions of each residue to the NanA–chlorogenic acid interaction [10,22].

The binding free energy of a protein-ligand complex in solvent can be given by [37]:

$$∆G_{bind}=∆G_{complex}-\left[∆G_{protein}+∆G_{lig}\right]$$

In the formula above, the Δ*G_complex_* is the total free energy of the complex, and the Δ*G_protein_* and Δ*G_lig_* represent energies of the isolate protein and ligand, respectively. 

The MM-PBSA method can be conceptually summarized as:

$$∆G_{bind}=∆E_{gas}+∆G_{sol}=∆E_{vdw}+∆E_{ele}+∆G_{polar}+∆G_{nonpolar}$$

∆*E_gas_* is the average molecular mechanics potential energy in a vacuum (i.e., gas-phase energy), which includes van der Waals (∆*E_vdw_*) and electrostatic (∆*E_ele_*) interactions; ∆*G_solv_* denotes contribution to the solvation free energy that consists of polar solvation (∆*G_polar_*) and nonpolar solvation (∆*G_nonpolar_*) energies [37].

## 5. Conclusions

In the current study, the binding mechanism of chlorogenic acid, a potential *S. pneumoniae* inhibitor, was explored by molecular docking, molecular dynamics simulation, and free energy calculation. The results indicate that chlorogenic acid might establish the interaction with *S. pneumoniae* NanA via hydrogen bonds, salt bridge, and cation-π. The interaction residues involved Arg347, Ile348, Lys440, Asp372, Asp417, and Glu768. It is noteworthy that the location of the side chain of Arg347 might form a cap-like structure to hold on to the benzene ring of chlorogenic acid. The results from total binding energy calculation indicated that chlorogenic acid had a strong binding potential with NanA, and that the electrostatic interactions were dominant in the total energy contribution. The results in this study will be useful for the development of efficient drug targets and provide a theoretical basis for the mechanism of new *S. pneumoniae* inhibitors.

## Figures and Tables

**Table 1:** Hydrogen bond occupancies for the NanA–chlorogenic acid complex.

Acceptor		Donor		Presence (%)
Chlorogenic acid	O7	Arg347	N-H	19.6%
Chlorogenic acid	O8	le348	N-H	58.4%
Asp372	COO-	Chlorogenic acid	O-H	78.4%
Chlorogenic acid	O2	Asp417	O-H	67.3%
Glu768	COO-	Chlorogenic acid	O-H	99.9%

**Table 2:** Calculation of binding free energy using molecular mechanics Poisson–Boltzmann surface area (MM-PBSA).

Energy Components	Values (kJ/mol)
Van der Waals energy	−140.06 ± 15.42
Electrostatic energy	−953.77 ± 32.38
Polar solvation energy	282.28 ± 25.02
SASA energy	−17.89 ± 0.78
Binding energy	−829.44 ± 19.31

## Supplementary Materials

Supplementary materials can be found at https://www.mdpi.com/1422-0067/21/3/1003/s1.
