# Supplementary material for: Exploration of Binding Mechanism of a Potential Streptococcus pneumoniae Neuraminidase Inhibitor from Herbaceous Plants by Molecular Simulation
Source: Int J Mol Sci. 2020 Feb 3;21(3):1003. doi: 10.3390/ijms21031003 (PMC7038148; doi:10.3390/ijms21031003)
Supplement: Supplementary file 1 [file ijms-21-01003-s001.pdf]

Exploration of binding mechanism of a potential Streptococcus pneumoniae neuraminidase inhibitor from herbaceous plants by molecular simulation

Shanshan Guan [1,2*], Ketong Zhu [1,2], Yanjiao Dong [1,2], Hao Li [1,2], Shuang Yang [1,2], Song Wang [3], Yaming Shan [4*]

1 College of Food Engineering, Jilin Engineering Normal University, Changchun, Jilin 130052, China

2 Key Laboratory of Molecular Nutrition at Universities of Jilin Province, Changchun, Jilin 130052, China

3 Laboratory of Theoretical and Computational Chemistry, Institute of Theoretical Chemistry, Jilin University, Changchun 130023, China

4 National Engineering Laboratory for AIDS Vaccine, School of Life Sciences, Jilin University, Changchun, Jilin 130012, China

* Corresponding authors at:
College of Food Engineering, Jilin Engineering Normal University; Key Laboratory of Molecular Nutrition at Universities of Jilin Province, Changchun, Jilin, China.
(Guan Shanshan) (email: guanshanshan@jlenu.edu.cn);
National Engineering Laboratory for AIDS Vaccine, School of Life Sciences, Jilin University, Changchun, Jilin 130012, China.(Shan Yaming).
(email: shanym@jlu.edu.cn)

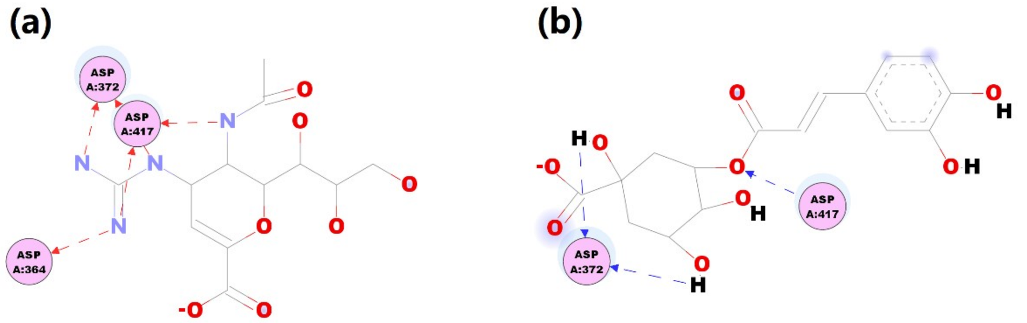

Fig. S1 The detail difference of binding mode between (a) zanamivir and (b) chlorogenic acid

```
     320       330       340       350       360       370       380       390
....|....|....|....|....|....|....|....|....|....|....|....|....|....|....|....|.
LPEGAALTEKTDIFESGRNGKPNKDGIKSYRIPALLKTDKGTLIAGADERRLHSSDWGDIGMVIRRSEDNGKTWGDRVTI

     400       410       420       430       440       450       460       470
...|....|....|....|....|....|....|....|....|....|....|....|....|....|....|....|.
TNLRDNPKASDPSIGSPVNIDMVLVQDPETKRIFSIYDMFPEGKGIFGMSSQKEEAYKKIDGKTYQILYREGEKGAYTIR

     480       490       500       510       520       530       540       550
...|....|....|....|....|....|....|....|....|....|....|....|....|....|....|....|.
ENGTVYTPDGKATDYRVVVDPVKPAYSDKGDLYKGNQLLGNIYFTTNKTSPFRIAKDSYLWMSYSDDDGKTWSAPQDITP

     560       570       580       590       600       610       620       630
...|....|....|....|....|....|....|....|....|....|....|....|....|....|....|....|.
MVKADWMKFLGVGPGTGIVLRNGPHKGRILIPVYTTNNVSHLNGSQSSRIIYSDDHGKTWHAGEAVNDNRQVDGQKIHSS

     640       650       660       670       680       690       700       710
...|....|....|....|....|....|....|....|....|....|....|....|....|....|....|....|.
TMNNRRAQNTESTVVQLNNGDVKLFMRGLTGDLQVATSKDGGVTWEKDIKRYPQVKDVYVQMSAIHTMHEGKEYIILSNA

     720       730       740       750       760       770       780       790
...|....|....|....|....|....|....|....|....|....|....|....|....|....|....|...
GGPKRENGMVHLARVEENGELTWLKHNPIQKGEFAYNSLQELGNGEYGILYEHTEKGQNAYTLSFRKFNWDFLSKDL
```

Fig. S2 The sequence of NanA

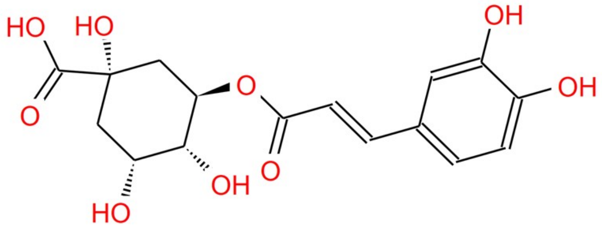

Fig. S3 The structure of Chlorogenic acid

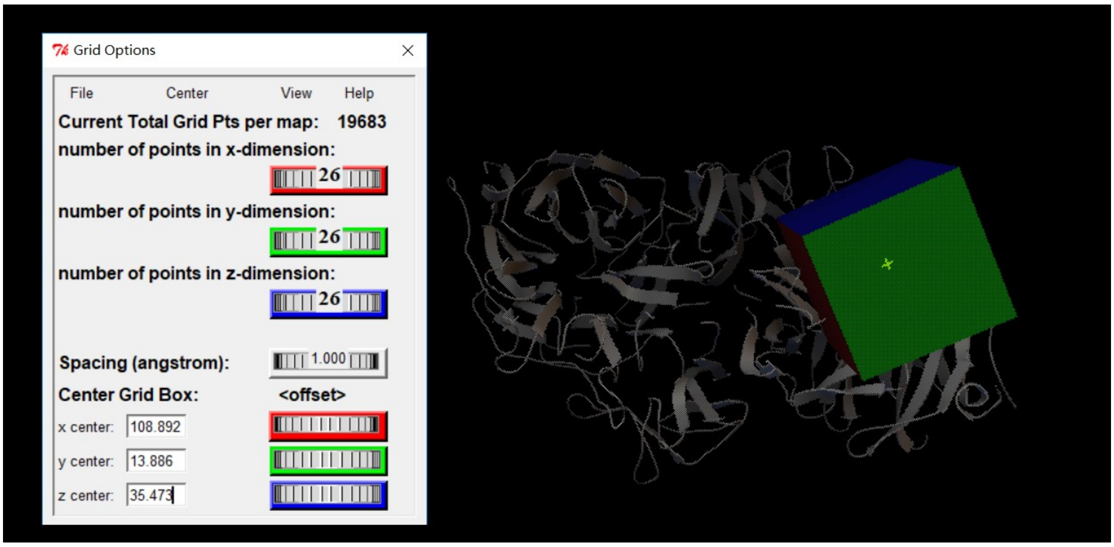

Fig. S4 The grid detail used in the docking simulation

Tab. S1 Protonation states for titratable residues of NanA

Protonated residues in complex	
ChainA	ChainB
all Arg, all Lys; Asp417, Asp434, Glu620, His612, His 634	all Arg, all Lys; Asp364 Asp417, Asp434, His612, His634
